# Recent advances in understanding necrotizing enterocolitis

**DOI:** 10.12688/f1000research.17228.1

**Published:** 2019-01-25

**Authors:** Mashriq Alganabi, Carol Lee, Edoardo Bindi, Bo Li, Agostino Pierro

**Affiliations:** 1 Division of General and Thoracic Surgery, Translational Medicine Program, The Hospital for Sick Children, Toronto, Ontario, Canada

**Keywords:** necrotizing enterocolitis (NEC), pathophysiology, premature, neonates

## Abstract

Necrotizing enterocolitis is a devastating intestinal disease affecting preterm infants. In spite of ongoing research and advancement in neonatal care, mortality remains high, especially in infants with advanced disease. The mechanism of disease development, the progression of intestinal injury, and management remain areas of ongoing research and controversy. In this review, we examine our current understanding of the disease, its epidemiology, the risk factors associated with the development of the disease, and its pathophysiology. We also describe current management and new emerging research highlighting potential future directions.

## Introduction

Necrotizing enterocolitis (NEC) is an inflammatory intestinal disease that affects 5–7% of preterm neonates ^1^. It is characterized by variable intestinal injury from epithelial injury to transmural involvement and perforation. It is also marked by inflammation and often bacterial invasion ^2^. NEC is one of the leading causes of morbidity in preterm infants ^3^. It affects nearly 10% of preterm infants with a birth weight of <1,500 grams ^4^. The mortality rate for preterm infants who have extremely low birth weight (<1,000 grams) is 30–50% and for infant with a very low birth weight (VLBW) (<1,500 grams) is 10–30%, and there has not been a significant change in the past 20 years ^5^.

To assess the severity of NEC, Bell’s classification was proposed in 1978. It categorizes the severity of NEC based on clinical and radiographic signs and remains the most widely used tool in early assessment. In recent years, however, this staging criterion has been modified as our understanding of the disease has improved, yet there continues to be controversy about the validity of this staging system at lower gestational ages (GAs) ^6^. Severity of NEC plays a key role in both the management and the outcome of affected neonates. Neonates with proven or advanced NEC, categorized as Bell’s stage II and III, respectively, are at risk of developing peritonitis, sepsis, bowel perforation, and other severe systematic complications including capillary leak syndrome and multi-system organ failure ^7^.

The pathophysiology of NEC is multifactorial and remains not fully understood. The risk factors for the development of the disease are multiple and some are controversial. This leads to difficulty in establishing novel strategies to prevent the development of NEC and its progression. Herein we discuss some of our latest understanding of the disease’s epidemiology, risk factors, pathophysiology, treatment strategies, and future directions.

## Epidemiology

The incidence of proven NEC (Bell’s stage II and III) in preterm babies depends on both the degree of prematurity and the geographic location of the patient. A recent systematic review on the incidence of NEC in high-income countries found variation in the incidence of the disease based on GA, birth weight, and country ^8, 9^. Overall, NEC incidence was highest among the most preterm infants. In infants born at a GA of <28 weeks, the lowest reported incidence of NEC was in Japan (2%) and the highest in Australia, Canada, and Italy (7–9%) ^8^. In neonates with a GA of between 28 and 31 weeks, reported NEC incidence was also lowest in Japan (0.2%), while other developed nations had incidence rates ranging from 2–3%. Similarly, for VLBW infants, NEC incidence ranged from 2% in Japan to 6–7% in the USA and 9% in Poland ^8– 10^. These findings collectively indicate that the degree of prematurity and low birth weight are important factors in developing NEC. The varied incidence rates between countries suggest various factors influencing the development of NEC including environment, diet, and genetic predisposition.

## Risk factors

The only consistently described risk factors for NEC are formula feeding, intestinal dysbiosis, low birth weight, and prematurity ^11^. Low birth weight and prematurity are the most commonly reported risk factors for NEC, with the lowest birth weights and GAs having the highest incidence of NEC ^12^. Maternal factors such as chorioamnionitis, cocaine abuse, in-utero growth restriction, increased body mass index, intrahepatic cholestasis during pregnancy, lack of prenatal steroids, mode of delivery, placental abruption, preeclampsia, and smoking have inconsistently been implicated in the development of NEC ^13– 17^. In addition, many other risk factors for the development of NEC have been reported including administration of acid-suppressing medications, acute hypoxia, antibiotic exposure, blood transfusions, cardiac anomalies, neonatal anemia, and poor intestinal perfusion ^18– 21^. Finally, prolonged use of indomethacin to promote the closure of patent ductus arteriosus (PDA) has been shown to be associated with the development of NEC ^22, 23^. However, the incidence of NEC was not lower in infants who underwent primary surgical closure of PDA compared to infants treated with indomethacin ^24^ ( Table 1).

**Table 1.** Risk factors for the development of necrotizing enterocolitis.

**Maternal factors** Chorioamnionitis Cocaine abuse In-utero growth restriction Increased body mass index Intrahepatic cholestasis during pregnancy Lack of prenatal steroids Mode of delivery Placental abruption Preeclampsia Smoking
**Main risk factors** Low birth weight Prematurity Formula feeding Intestinal dysbiosis
**Other risk factors** Acid-suppressing medications Acute hypoxia Antibiotic exposure Blood transfusions Cardiac anomalies Neonatal anemia Poor intestinal perfusion Prolonged use of indomethacin for patent ductus arteriosus closure

## Pathophysiology

NEC affects multiple organs, and its pathophysiology appears to be multifactorial. The classical understanding of NEC pathophysiology suggests that intra-luminal bacteria disrupt and invade the intestinal epithelium at the tips of intestinal villi ^25^. Endotoxin from these bacteria binds to Toll-like receptor 4 (TLR4) found on the intestinal epithelial cells, activating pathogen-associated molecular pattern (PAMP) receptors, which facilitate the breakdown of the gut barrier and allow bacteria to translocate ^26^. This process subsequently leads to an intense inflammatory response in the lamina propria mediated by tumor necrosis factor-alpha (TNF-α), interleukin-1β (IL-1β), and other inflammatory cytokines ^27^. Vasoactive substances are also released in the intestine, and those associated with NEC include platelet-activating factor (PAF), endothelin-1 (ET-1), and nitric oxide (NO) ^28^. Intestinal inflammation also activates complement and coagulation systems. In these systems, leukocytes and platelets adhere to the endothelium, preventing blood flow in the microvascular structure of the small intestine and leading to tissue injury. Additional damage to the endothelium from adherent neutrophils and platelets also impairs NO generation needed for vasorelaxation ^29, 30^. Through the efforts of various research laboratories, including ours, potential mechanisms have been explored to investigate how the disease may develop and how it may be treated ( Table 2).

**Table 2.** Factors involved in necrotizing enterocolitis pathophysiology.

Nitric oxide
Toll-like receptor 4
Microvascular blood flow (intestinal ischemia)
Dysbiosis
Reduced activity of intestinal stem cells

### Nitric oxide

The pathogenesis of NEC is likely initiated by postnatal insults on the immature intestine in the presence of some of the previously mentioned risk factors. These factors lead to the initial epithelial injury, which causes an intestinal inflammatory response and release of inflammatory mediators ^31^. Ford *et al*. ^32^ first highlighted the role of NO and that of inducible NO synthase (iNOS)-derived NO in NEC development. NO plays a critical role in NEC development, and the details of its role have been extensively explored. Once intestinal barrier failure occurs, the lamina propria is exposed to increased levels of endotoxins and other bacterial products owing to bacterial translocation ^33^. The net result is activation of the neonatal immune system, which triggers an inflammatory cascade that leads to a severe pro-inflammatory response characterized by the release of NO, cytokines, and prostanoids. NO interacts with superoxide and leads to the production of peroxynitrite, a potent oxidant ^34^. This subsequently leads to enterocyte apoptosis or necrosis as well as impairment of both enterocyte proliferation and epithelial repair through enterocyte migration. The imbalance between tissue injury and repair further catalyzes the inflammatory cascade involved in NEC development. The ultimate consequence of these insults is further epithelial injury with risk for bacterial translocation resulting in sepsis, a vicious cycle which can lead to severe inflammation, bowel necrosis, perforation, and death.

### Toll-like receptor 4

The premature infant intestine is characterized by elevated expression of TLR4 on the intestinal epithelium ^35, 36^. TLRs play an essential role in the activation of innate immunity by recognizing specific patterns of microbial components ^37^. TLR4 expression was increased in mice and humans with NEC ^38, 39^, and mutations in the TLR4 signaling pathways have been described in human NEC ^40– 42^. Additionally, TLR4 knockout mice were protected from NEC induction ^43^. TLR4 is activated by lipopolysaccharides on Gram-negative bacteria ^36^. Activation of TLR4 by intestinal lumen microbes results in barrier injury and impaired intestinal repair ^39^, which consequently allows the translocation of the luminal bacteria, vasoconstriction, intestinal ischemia, and NEC ^44^. TLR4 can also be inhibited by probiotic bacteria that activate TLR9 ^45^ and can prevent goblet cell differentiation ^43^, which are needed to maintain the physical mucous intestinal barrier to pathogenic bacteria.

### Microvascular blood flow

NEC is a disease often characterized by areas of intestinal ischemia with insufficient blood supply. The development of NEC has been associated with generalized neonatal hypoxia and exchange transfusions ^46^. Derangement of the intestinal microcirculation in NEC leads to areas of poor blood flow which may help facilitate the inflammatory cascade, resulting in intestinal injury ^47^. Feeding and postprandial hypoxia have been shown to synergistically induce intestinal hypoxia in experimental NEC, highlighting the important balance between oxygen supply and demand ^48^. The role of circulation in the pathogenesis of NEC continues to be a topic of research interest.

### Microbiota

Dysbiosis is a disruption of gut microbiota development and its homeostasis, which has been associated with the development of NEC ^49^. This pathological process involves a lack of beneficial commensal microbes combined with a low diversity of bacteria, which allows the overgrowth of pathogenic bacteria that induce an inflammatory response ^50^. A meta-analysis of intestinal dysbiosis in preterm infants preceding NEC found an increased relative abundance of Proteobacteria and a decreased relative abundance of Firmicutes and Bacteroides ^51^. Dysbiosis has been associated with the use of antibiotics and/or antacids in the NICU, formula feeding, and inflammatory response dysregulation ^52^. NEC and matched control fecal samples tested for bacterial diversity and clustering showed that NEC patients tend to have less-diverse microbiomes and different distributions of the intestinal bacteria ^53– 56^. Proteobacteria may trigger a strong inflammatory response and colonization by anaerobic bacteria, which have been associated with NEC ^57^. However, since Proteobacteria are also common constituents in the intestinal microbiome of preterm neonates who do not develop NEC, the role of microbiota remains unclear and represents only part of the complex pathogenesis ^58^. While prophylactic probiotics have been shown to reduce the incidence of NEC when baseline incidence levels are high ^59^, not all probiotic preparations are equally efficacious ^60^. Comparing the efficacy of different strains, mixtures of multiple strains and long-term safety remain areas of current research ^61, 62^.

### Intestinal stem cells and epithelial regeneration

The small intestinal epithelium renews every three to six days, a rate driven by vigorous proliferation within the intestinal crypts towards the villus tip ^63^. Within the crypts there is a distinct stem cell zone containing intestinal stem cells (ISCs). ISCs are responsible for producing progenitor cells that differentiate into various types of epithelial cells including enterocytes, goblet cells, entero-endocrine cells, and Paneth cells ^64^. ISCs are activated and replicate in response to intestinal injury. To measure the number of ISCs, leucine-rich repeat-containing G-protein-coupled receptor 5 (Lgr5), a well-established wingless integrated (Wnt)-associated stem cell marker ^65^, has been used. NEC has been characterized by a decrease in Lgr5-positive ISCs ^63, 66^, and restoration of ISC activity appears to have a beneficial effect ^66^. The mechanism by which ISC viability is disrupted in NEC remains poorly understood and is currently being investigated.

## Treatment strategies

### Preventative treatments

Breast feeding has been shown to reduce the incidence of NEC relative to formula feeding ^59, 67^. In examining which components of breast milk help in reducing NEC incidence, breast milk-derived exosomes ^68^ and human milk oligosaccharides (HMOs) ^69^ were investigated using an experimental model of NEC. Breast milk-derived exosomes have been shown to promote intestinal epithelial cell viability and stimulate intestinal stem cell activity ^68^. HMOs increased the number of goblet cells and mucin expression, stabilizing the intestinal barrier ^69^. In infants with a birth weight of <1,500 grams, the use of prophylactic probiotics reduced the incidence of NEC (RR 0.34, 95% CI of 0.23–0.50) ^70^, the risk of NEC-associated mortality (RR 0.56, 95% CI of 0.34–0.93) ^71^, and the total length of hospital stay ^72^. Notably, however, probiotic administration did not significantly reduce the risk of developing NEC in infants with a birth weight of <1,000 grams ^73^ or the risk of developing NEC requiring surgery in infants of any size (RR 0.56, 95% CI of 0.56–1.25) ^70^. Despite remaining uncertainties, the American Pediatric Surgical Association (APSA) Outcomes and Clinical Trials Committee Cochrane Review supports the prophylactic use of probiotics in preterm infants with a birth weight of <2,500 grams to reduce the risk of NEC in addition to the use of human breast milk rather than formula whenever possible ^70^.

### Medical and surgical treatments

Suspicion of NEC is frequently based on clinical presentation, which can include feeding intolerance, abdominal distention, bloody stools, emesis, and gastric retention. These signs lead to further workup including blood work to detect potential thrombocytopenia or metabolic acidosis and imaging studies to identify dilated loops of bowel, intestinal perforation, pneumatosis intestinalis, and portal venous gas. Depending on imaging findings and clinical presentation, surgical intervention is considered. Abdominal X-ray and ultrasound have been shown to be useful in helping to monitor the progression of the disease and detecting the presence of NEC ^74^. In general, for Bell stage I (suspected NEC), supportive medical management alone is provided. For Bell stage II (proven NEC), medical management is usually tried first. This includes antibiotic treatment, nasogastric decompression, and total parenteral nutrition. If the patient fails to respond to medical treatment, surgical management is considered ^75^. Patients with Bell stage III (advanced NEC) can be treated medically and may require inotropic support. However, neonates who develop intestinal perforation, have suspected bowel necrosis, or fail to respond to medical treatment require surgical treatment. Among VLBW infants, 27–52% require surgical intervention ^76^.

## Future directions

Current research using both animal models and human tissue has yielded novel potential therapeutic avenues ( Table 3). For example, the prospects of using stem cell therapy ^77^ and breast-milk derived exosomes ^68^ appear to be promising. Amniotic fluid stem cells given by intraperitoneal injection migrated to the intestinal villi and colonized almost exclusively the damaged intestine of NEC rat pups, where they promoted auto-regeneration of the intestinal epithelium ^78^. Stem cell-derived exosomes have also reduced the incidence and severity of experimental NEC ^79^. In addition, milk-derived exosomes have recently been shown to reduce intestinal epithelial injury ^68^. Safety and efficacy trials still need to take place before some of these potential treatments may become clinically available. Considering the lack of improvement and the unchanged mortality associated with NEC, it is promising to see that several avenues are being explored in both disease prevention and treatment.

**Table 3.** Future potential therapy for necrotizing enterocolitis.

Breast milk component (human milk oligosaccharides or exosomes) administration
Prophylactic probiotics
Stem cell administration
